# Wireless transmission of biosignals for hyperbaric chamber applications

**DOI:** 10.1371/journal.pone.0172768

**Published:** 2017-03-15

**Authors:** Carlos Perez-Vidal, Luis Gracia, Cristian Carmona, Bartomeu Alorda, Antonio Salinas

**Affiliations:** 1 Department of Systems Engineering and Automation, Miguel Hernandez University, Elche, Spain; 2 Instituto IDF, Universitat Politècnica de València, Valencia, Spain; 3 Department of Physics, Illes Balears University, Palma, Spain; 4 MEDIBAROX Hyperbaric Unit, Perpetuo Socorro Hospital, Alicante, Spain; Tongji University, CHINA

## Abstract

This paper presents a wireless system to send biosignals outside a hyperbaric chamber avoiding wires going through the chamber walls. Hyperbaric chambers are becoming more and more common due to new indications of hyperbaric oxygen treatments. Metallic walls physically isolate patients inside the chamber, where getting a patient’s vital signs turns into a painstaking task. The paper proposes using a ZigBee-based network to wirelessly transmit the patient's biosignals to the outside of the chamber. In particular, a wearable battery supported device has been designed, implemented and tested. Although the implementation has been conducted to transmit the electrocardiography signal, the device can be easily adapted to consider other biosignals.

## Introduction

Hyperbaric medicine, also known as Hyperbaric Oxygen (HBO2) therapy, is the medical use of 100% pure oxygen in a pressurized chamber, called the hyperbaric chamber, at pressures from 1.4 to 2.5 atmospheres, as specified by the UHMS (Undersea and Hyperbaric Medical Society), the world's leading reference in Hyperbaric Medicine [1][2]. The number of HBO2 therapies is increasing every year [3] [4] [5]. For this purpose, Hyperbaric Chambers (HC) or diving chambers are used. A HC consists of a sealed vessel with a forced supply of air to increase the pressure inside it. The main indications of this therapy are (amongst others): Air or Gas Embolism; Carbon Monoxide Poisoning; Clostridial Myositis and Myonecrosis (Gas Gangrene); Crush Injury, Compartment Syndrome and Other Acute Traumatic Ischemias and; Decompression Sickness. The complete list of indications can be found in [6].

The list of indications is growing as new properties of the therapy are demonstrated and accepted by the scientific and medical community. The number of patients receiving HBO2 treatment is constantly increasing, therefore new systems and procedures are required to improve the security, efficiency and performance of medical instrumentation. To increase the HBO2 treatment versatility and to decrease the number of cables through HC walls, this paper presents the results of a ZigBee communication platform to establish a wireless channel.

**Fig 1:**
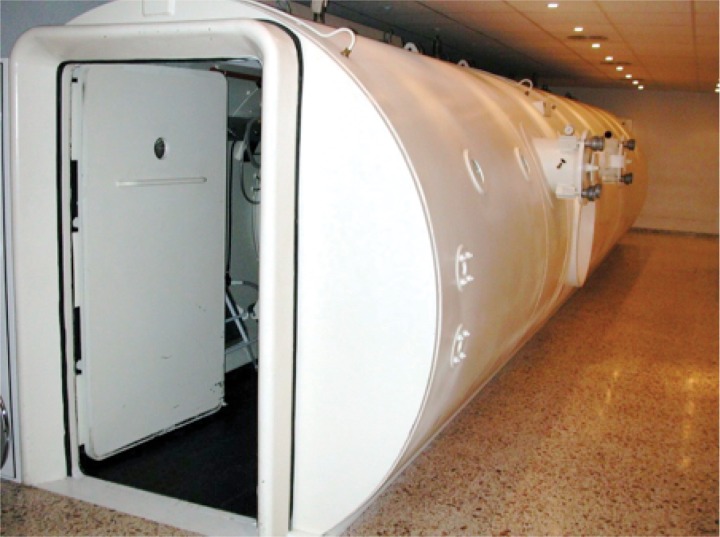
Multiplace Hyperbaric Chamber used in this work: Outside photo.

**Fig 2:**
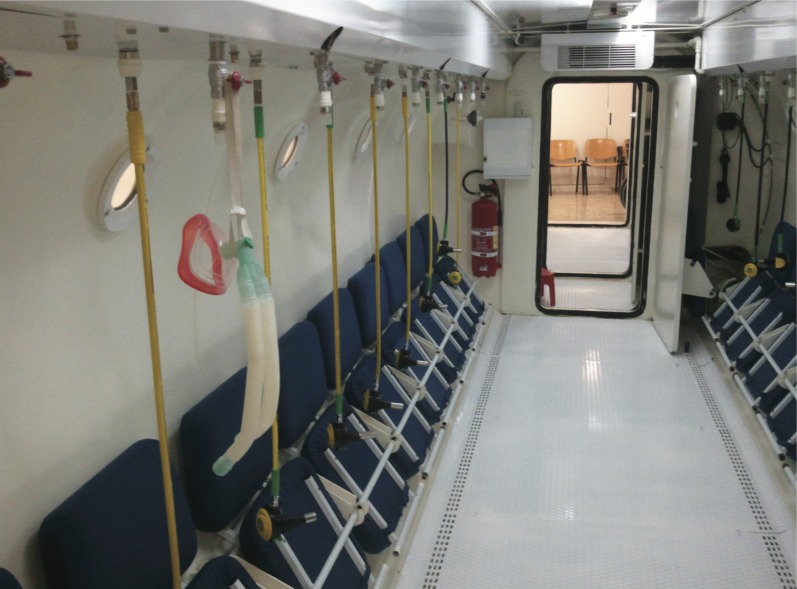
Multiplace Hyperbaric Chamber used in this work: Inside photo.

**Fig 3:**
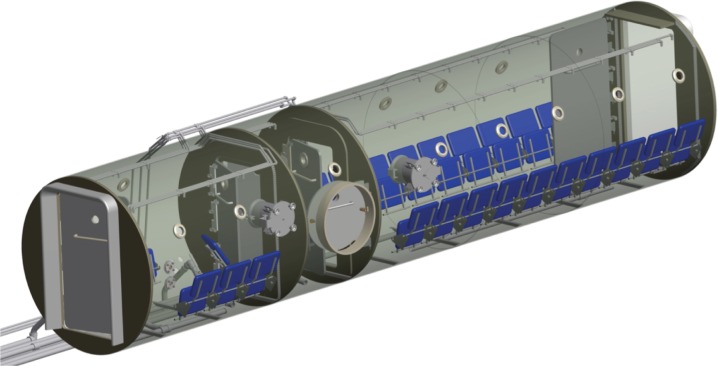
Multiplace Hyperbaric Chamber used in this work: Partly transparent 3D model of the chamber.

The solution proposed in [7] deals with this issue using a peer-to-peer communication architecture. These types of approaches lack traffic network monitoring and management capabilities and, therefore are not suitable when the number of emitters and receivers dramatically grows. In these cases, with a large number of patients being monitored simultaneously, the system produces interferences reducing the radio link capacity. Other solutions like [8] use Bluetooth communication for wireless transmission of a patient’s vital signs, where a network architecture is used with a short-range radio transceiver that is not suitable for HCs since the communications capability outside the vessel is limited. In this sense, a commercial device based on Bluetooth technology has been evaluated in this study to check the coverage area. In particular, the Shimmer Wireless Electrocardiography Sensor [9] was tested in the Perpetuo Socorro HC (see Figs 1–3), but the signal coverage was extremely reduced and the receiver had to be installed, fixed to one of the HC windows in order to establish the communication.

Bluetooth based solutions [10] [11] are mainly used for home automation networks in which long-term monitoring is needed. Finally, the system proposed in [12] is a ZigBee (IEEE802.15.4) based communication platform working on *ad-hoc* hardware. Section 2 analyses the most popular communication technologies.

The system designed and implemented in this study fits for the application of the HC vital signs transmission with the following special considerations taken into account. Firstly, a high number of patients will be monitored and therefore, the number of wireless nodes will be higher than in other applications. Secondly, high interference levels between emitters and receivers due to metal walls are expected. Thirdly, the high-pressure conditions force the use of a special enclosure to protect the system conditioning the ventilation and power consumption.

After the analysis of commercial solutions and taking into account the low coverage area obtained with Bluetooth communication, a self-solution has been developed in this study to solve the particular problem of the Perpetuo Socorro Hospital hyperbaric chamber located in Alicante, Spain. This paper proposes a low power consumption battery based system using ZigBee communication [13] to transmit a patient’s vital signs. An example focused on the electrocardiography (ECG) signal acquired using three electrodes is given, but the system could be used to transmit other electrical biosignals by making very few changes. In addition, other non-electrical biosignals could be transmitted performing some other changes in the proposed platform.

The battery-based solution proposed in this paper can be considered as a wearable device due to its reduced size. It allows having one device per patient in case of necessity, avoiding bulky equipment and/or uncomfortable cables attaching the patient to the HC. A ZigBee network has been selected to implement the platform due to its reception coverage area. In addition, up to 65k nodes can be incorporated to the network and low-power features are especially suitable. Furthermore, the ZigBee network can use router nodes to expand the coverage area or introduce several topologies depending on application needs.

The paper is organized as follows. Section 2 makes a comparison of wireless communication technologies to choose the best option for this specific application. This decision is supported by a state of the art review. Section 3 presents the biosignals that could be monitored and transmitted. It has information about each biosignal, the special features of the HC environment and a block diagram with general information of the system. Section 4 is focused on a specific implementation case. For the ECG signal, detailed schemes for implementation are given. Section 5 presents the results obtained in this work and Section 6 summarizes the conclusions.

## Comparison of wireless communication technologies

This section comparatively analyses the characteristics of the main standards for wireless communication in home or personal area networks (i.e., in short range smart grid applications [14]) in order to select the most suitable one for this work. In particular, the following standards are considered: *Wi-Fi* (IEEE 802.11a/b/g), *Bluetooth* (IEEE 802.15.1) and *ZigBee* (IEEE 802.15.4). Note that, other popular standards like UWB (IEEE 802.15.3) or WiMAX (IEEE 802.16) are out of the scope of the present application since they are designed for particularly large bandwidths (up to 480 Mbps [15]) and coverage areas (a metropolitan area network of up to 48 km [16]), respectively. Table 1 summarizes the main differences between the three mentioned standards. This table has been obtained combining several sources [14–20]. Next, the main features of the three standards are analyzed:

- Coexistence Mechanism: Since Bluetooth, ZigBee and Wi-Fi use the same frequency band, the issue of interference must be dealt with. In particular, Bluetooth uses adaptive frequency hopping to avoid channel collision, while ZigBee and Wi-Fi provide dynamic frequency selection and transmission power control.
- Security: All three protocols have encryption and authentication mechanisms; Bluetooth uses the E0 stream cipher and shared secret with 16-bit cyclic redundancy check (CRC); ZigBee uses the advanced encryption standard (AES) block cipher with counter mode (CTR); Wi-Fi uses the RC4 stream cipher for encryption and the CRC-32 checksum for integrity.
- Network Size: The maximum number of devices belonging to the network's building cell is 8 for Bluetooth Piconet, over 65000 for a ZigBee star network, and 2007 for a structured Wi-Fi basic service set (BSS).
- Transmission time: This depends on the data rate, the message size and the distance between two nodes. In general, the transmission time for the ZigBee is significantly longer than the others due to the lower data rate (250 Kbs). Therefore, ZigBee is specially designed for small messages.
- Coverage area: ZigBee and Bluetooth are intended for a personal area network (about 10m), whereas Wi-Fi is oriented to WLAN (about 100m). However, ZigBee can also reach 100m in some applications.
- Power Consumption: ZigBee and Bluetooth are intended for portable products so have very low power consumption for long battery lives, whereas Wi-Fi is designed for a longer connection and devices with a substantial power supply. It is interesting to remark that, the low power consumption battery based technique is also instrumental for network robustness/connectivity in general [21].
- Latency: ZigBee has a much lower latency compared to Bluetooth and Wi-Fi. For instance, a Bluetooth device may take approximately three seconds to wake up while a Zigbee device may only take a few milliseconds [17].

**Table 1:** Comparison between wireless solutions: ZigBee, Bluetooth and Wi-Fi.

	*ZigBee* (IEEE 802.15.4)	*Bluetooth* (IEEE 802.15.1)	*Wi-Fi* (IEEE 802.11a/b/g)
Range	10–100 meters	10 meters	50–100 meters
Bandwidth	250 Kbps	1 Mbps	54 Mbps
Topology	Ad-hoc (star, three or mesh)	Ad-hoc (small networks)	Point to access point
Frequency	• 868 MHz (Europe) • 900–928 MHz (North America) • 2.4 GHz (rest of the world)	2.4 and 5 GHz	2.4 GHz
Coexistence	Dynamic frequency selection	Adaptive frequency hopping	Dynamic frequency hopping
Power^a^ TX/RX/Standby	25 mA / 27 mA / 0.003 mA	57 mA / 47 mA / 0.2 mA	219 mA /217 mA / 20mA
Typical applications	Industrial control sensor networks	Internet access	Headsets, file transfer

^a^Data values extracted from [15].

Note that these three standards are neither complementary nor competitors, but just essential standards for different targeted applications:

1. Wi-Fi is suited for higher data-rate applications over larger areas. In particular, it is the most accepted protocol for wireless in-home communications in order to connect to the Internet.
2. Bluetooth has become popular for wireless connections for voice, data and audio applications over short range. However, it supports a limited number of nodes that can be a serious constraint.
3. ZigBee is a cheap reliable solution [18] particularly suitable for wireless devices that do not need high data rates but that require: low power; simple network configuration and management; and large device arrays. In particular, ZigBee has become the main choice for home automation networks [16] in order to cope with sensor and control devices [19]. For instance, ZigBee is the main option used in medical care fields [20].

Therefore, taking into account the analysis above, this paper considers the ZigBee option for the wireless communication from the inside to the outside of the HC since it provides the advantages of: low cost, low power consumption, low latency, large device arrays, etc.

## Biosignals and equipment

### Electrical biosignals

Bioelectrical voltages of the human body are rarely deterministic [21][22]. Their magnitudes vary with time even when all factors that originate them remain constant. Values of the same biosignal can vary considerably between different subjects even when they are healthy. This means that the values can be very different for different people even if they are normal values [23]. Table 2 shows some biosignal values in which their voltages and bandwidth are specified [24].

**Table 2:** Voltage and bandwidth for the most common, electrical biosignals.

Signal	Voltage	Bandwidth (Hz)
ECG (electrocardiography)	0.5-4mV	0.01–250
EEG (electroencephalography)	5–300μV	DC-150
EGG (electroglottography)	10–10000μV	DC-1
EMG (electromyography)	0.1-5mV	DC-10000
EOG (electrooculography)	50–3500μV	DC-50
ERG (electroretinography)	0–900μV	DC-50

Taking the ECG signal as example and to avoid deformations greater than 10%, the *American Heart Association* [25] recommends a minimum bandwidth of at least 0.1Hz to 100 Hz, but depending on the pathology to be detected, it may be necessary to work with a higher bandwidth [23]. For this reason, the bandwidth shown in Table 2 for ECG is 0.01Hz to 250Hz. In all those cases, the signal is obtained through an electrode connected to the patient’s skin.

### The hyperbaric chamber

A hyperbaric chamber is basically an enclosure in which a patient or a group of patients are exposed to a pressure higher than the atmospheric one. Under this environment, patients breathe pure oxygen during the treatment. Figs 1–3 show two views of the multi-place hyperbaric chamber installed at Perpetuo Socorro Hospital (Alicante, Spain) to apply hyperbaric oxygen treatments. The walls of a hyperbaric chamber are usually made of steel. The thickness of the walls of the hyperbaric chamber shown in Figs 1–3 is 12mm which creates a “Faraday effect”, which causes electromagnetic insulation, blocking any electromagnetic signal. Fortunately, hyperbaric chambers usually have a group of small windows (with 40mm thickness methacrylate for the case of Perpetuo Socorro Hospital) that allow bidirectional wireless communication.

Regarding the atmosphere in which the electronic system will be working, the pressure inside the chamber is usually around 2.5 atmospheres and all the equipment introduced inside it has to be certificated to work in hyperbaric environments, as Directives 2006/95/EC, 93/42/EEC and 97/23/EC of the European Commission have established. In addition, for the case of a mono-place hyperbaric chamber, it is pressurized using O2. This environment is especially dangerous for the patient in case of fire due to the high concentration of oxygen. In this project, a Hammond 1554G box has been used to deal with all these features.

### Block diagram for the proposed wireless system

**Fig 4:**
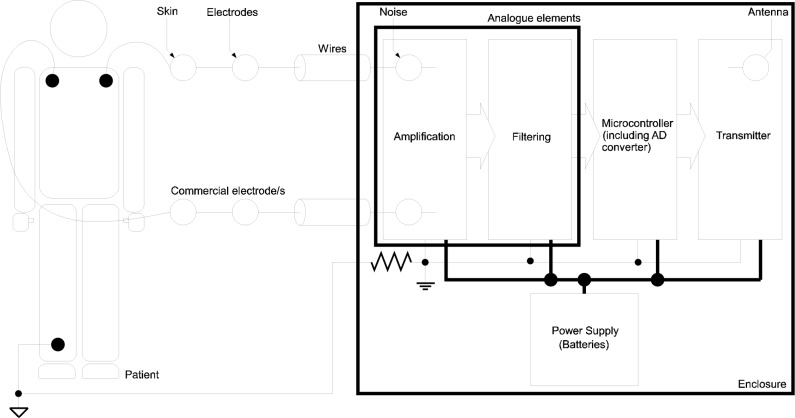
Block diagram of the proposed approach.

Electronic systems devoted to biosignal acquisition can be represented with a generic block diagram. For the proposed approach, this block diagram is shown in Fig 4, which depicts all the elements required for signal conditioning and wireless transmission. The diagram shows on the left side the electrodes attached to the patient’s skin. The electrodes acquire the voltage from the body of the patient and send it to an amplifier, with high common mode rejection and high input impedance. The amplifier, taking into account the biosignal voltage level (see Table 2), uses a gain in order to obtain a proper output voltage level. Subsequently, an analogue filter (either active or passive) rejects any frequency component out of the biosignal range. Then, this filtered analogue signal is transformed to digital by an analogue-to-digital converter, usually by means of special hardware inside a microcontroller. Finally, the resulting signal is transmitted wirelessly to avoid the use of cables through the hyperbaric chamber walls.

A power supply for the electronic device is also required. This power supply can be taken from the power line or from batteries. In the first case, two electrically isolated sources would be required in order to keep the patient insulated and safe from the power line. The block “amplification” would be input-output insulated as well.

### Non-electrical biosignals to be transmitted

The term biosignal refers to signals acquired from the human body. They can be electrical (e.g. ECG, EEG or any of the ones shown in Table 2) or non-electrical (e.g. respiratory signal, capnometry, temperature, etc.). The knowledge of this information plays an important role in medical diagnosis and treatments applied by clinicians and physicians. This paper is focused on electrical biosignals in which Fig 4 can be applied as an acquisition scheme. For the case of non-electrical biosignals, additional medical equipment is required to transform these biologic indicators into a voltage proportional to the physical magnitude that is being measured.

This medical equipment would replace the block named “Analogue elements” in Fig 4. In this case, a digital link (e.g. based on USB communication) could be established between the medical equipment and the “Microcontroller”. In addition, everything inside the chamber has to be certified for hyperbaric environments, or it must be introduced inside a special enclosure. In this case, the system might not be wearable due to the additional bulky equipment to be attached to the patient.

## Implementation for the ECG case

### Amplification and filtering

**Fig 5:**
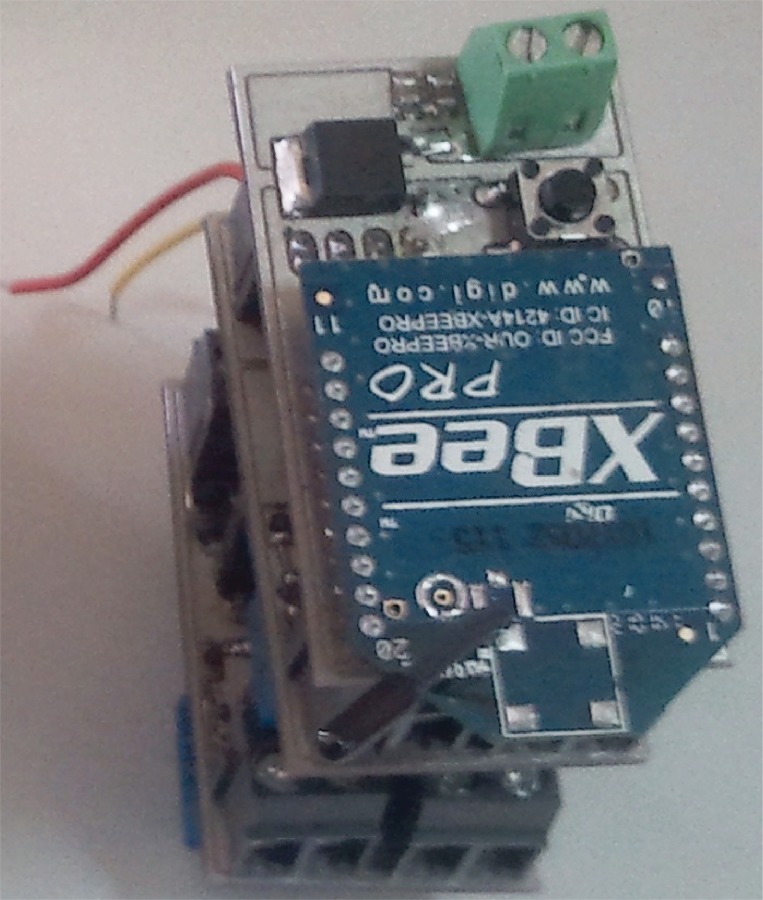
Electronic device for wireless transmission: Electronic components.

**Fig 6:**
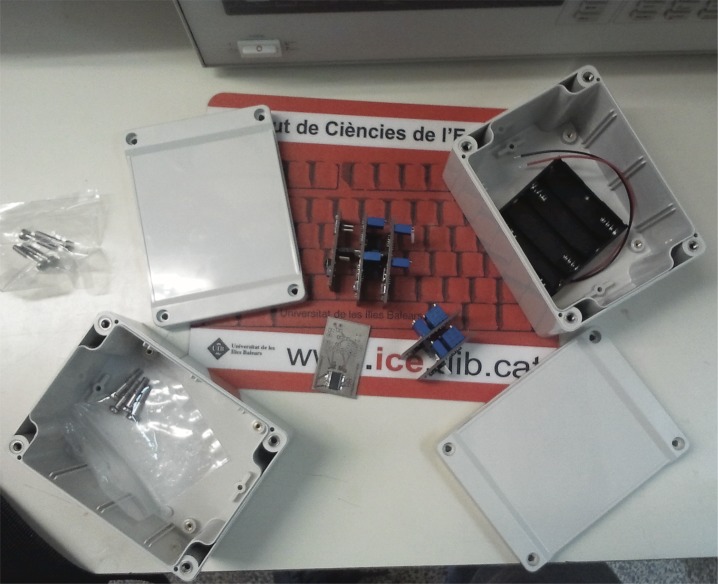
Electronic device for wireless transmission: Enclosure box and batteries.

This section presents a particular implementation of the block diagram shown in Fig 4 and the final device can be seen in Figs 5 and 6. Note that this implementation can be readily adapted to other electrical biosignals shown in Table 2, taking into account the corresponding voltages and bandwidths.

**Fig 7:**
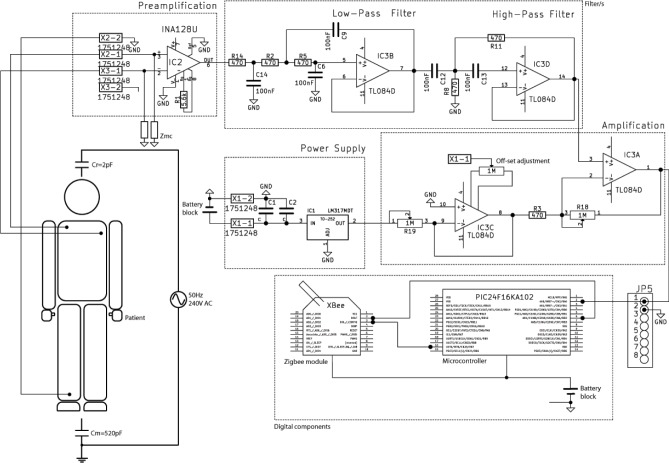
Implementation for the ECG case.

For measuring the ECG signal, Vermed A10005 Wet Gel electrodes have been used. As Microcontroller, a 16-bit Microchip PIC24F16KA102 processor has been used. This PIC has a 10 bit analogue to digital converter and is connected with a XBee Pro 2 module supplied by Digi International (http://www.digi.com). See Fig 7 for detailed information. The amplification and filtering blocks have been implemented as a “Pre-amplification then Filtering then Amplification” structure as can be seen in Fig 7. The pre-amplifier stage is designed to get a voltage gain of 10 with high CMRR and high noise rejection. The second stage is a low-pass Salen-Key filter with a cut-off frequency of 100Hz and damping ratio of 0,2 dB. The analogue final stage is a post-amplification using an operational amplifier with a voltage gain of 30.

Each stage is responsible for different functions:

- The pre-amplification stage eliminates the influence of some other physiological signals on the electrode like the voltage generated by muscle movements. This is achieved using a differential measurement between three points: two of them connected to the differential inputs of the amplifier and the last one used as reference node (see Figs 4 and 7 for more information).
- The filtering stage is able to remove the high frequency and low frequency noise components of the signal with no significant attenuation.
- The Amplification block amplifies the signal from ±5mV to ±1.5V (gain of 300) and adds an offset of 1.5V changing the output range from [-1.5…1.5V] to [0…3V].

### Microcontroller and ZigBee module configuration

The proposed system is based on a wireless ZigBee-based network to perform the management between the nodes inside the HC and a receiver module outside the HC. The external device acts as a coordinator node in the ZigBee network and is a real-time receiver module, which is plugged into a computer representing the HC control system.

**Fig 8:**
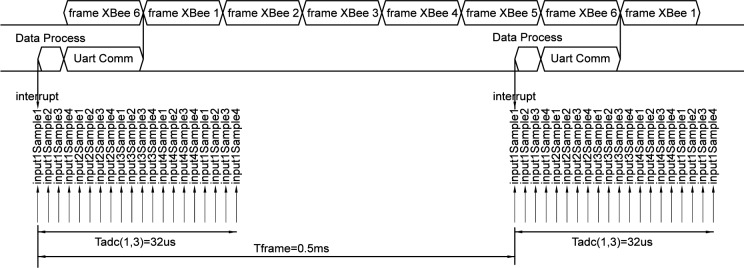
Transmission timing diagram.

The nodes inside the chamber perform not only the signal conditioning described in the previous section but also communication tasks and create the data frame to be transmitted to the external device. All these functions impact on system performance because they are time dependent. Hence, a precise time schedule must be defined to reduce unnecessary delays. Fig 8 shows the time diagram defined using an oversampling strategy [26] where Input(n) denotes each analogue input and Sample(n) the sample number (with n = 1, 2, 3 or 4). The proposed methodology establishes two different independent processes with different time configurations:

- The A/D conversion process, which defines the sampling frequency of each analogue input signal
- The communication process according to the maximum transmission rate

The timing pattern of the A/D conversion process is established considering that each analogue input signal is sampled four consecutive times at the minimum sampling period of 2μs (i.e., 500k samples per second). Therefore, considering four different analogue inputs, the same input signal is sampled using a conversion period TADC of 32μs, see Fig 8. As a result, 16 samples from 4 analogue signals are stored in the ADC buffer waiting to be transmitted. The continuous conversion strategy uses a rotation scheme that, after the sixteenth sample is stored (input4sample4 in Fig 8), a new rotation updates the A/D converter buffer with a new value (input1sample1 in Fig 8), then the second sample (input1sample2) and so forth. The samples stored in the A/D converter buffer are used to calculate the average value of the last four sampled-values for the same signal and, hence, smooth sample variability is achieved.

Beside the rotation conversion time described above, the program is working on communication tasks, i.e., defining the data frame and sending it to the wireless transmitter. The data frame is transmitted with a frequency rate of 2kHz. Each frame contains the average values of four vital signals. Fig 8 represents how the data frame is defined at each Tframe using an internal interruption generated at a fixed period, which acts as effective sampling frequency of four analogue signals. As a consequence, the transmission channel is divided on Tframe time windows where each node can send its data frame with no conflict with the other nodes sharing the same SSID network. The horizontal axis at the bottom of Fig 8 represents the time division of the transmission channel given by Tframe. There are two relevant issues about the continuous rotation sampling strategy:

- The sampling period TADC is shorter than transmission period Tframe. This sampling period minimizes the voltage differences between the four sample values of each analogue signal and can be considered sampled at the same transmission instant.
- The sampling process and the transmission process are executed independently in a continuous cycle. For this reason, the time delay between the four samples of each signal could not be equal. Hence, some samples could be converted at the present cycle and the rest at the previous cycle. This introduces an extra sampling delay between samples of 32μs (16 samples/500ksps). The averaging function reduces the impact of this extra sampling delay on signal reconstruction and smooths out the presence of conversion noise.

The time scheme showed in Fig 8 has been implemented at the node microcontroller. To perform the described time strategy, two different tasks (transmission and conversion) run in parallel. In this regard, the task has been designed using an interrupt-based programming strategy developing an event-based programming code. The main steps implemented in the microcontroller node are shown in Box 1.

**Box 1. Main steps of the interrupt-based code.**

**Initial Step**: *Configure peripheral hardware*

- Enable A/D converter for continuous conversion at 500 ksps.
- Configure 4 analogue input channels in the A/D conversion list.
- Configure Serial port function: 250kbps, 8 bits, non-parity, and one bit stop.
- Set Data_Send_Timer interrupt period to 0.5ms

**Main Step**: *Continuous A/D conversion using a 16 level ring buffer*. *Each analogue input channel is sampled four times and stored in four consecutive ring buffer positions*.

**Data_Send_Timer interrupt**: *Create a frame with the data*

- Average of last four samples stored for each analogue input channel.
- Sent the data frame with four healthy parameters values.
- Set a new Data_Send_Timer interrupt period.

The number of patients with node devices is an important feature of the proposed platform. The number of nodes that will communicate with the receiver module must take into account the broadband transmission mode and the time division of the transmission channel discussed above. In this regard, the wireless transmitter is configured to use the maximum transmission rate allowed by the ZigBee network, i.e., 250kbps. Hence, the maximum number of nodes is limited by the frame overhead and the frequency delay between each frame. Thus, considering that the broadcast mode reduces to minimum the frame overhead extension and a frequency delay between frames of 2kHz, the maximum number of patients with nodes is established in 6 patients per network. Therefore, using all the frequency channels, the maximum number of patients with continuous monitoring results in 96 patients (16 x 6). This number is large enough since the number of available seats in the HC is below that number.

## Results

**Fig 9:**
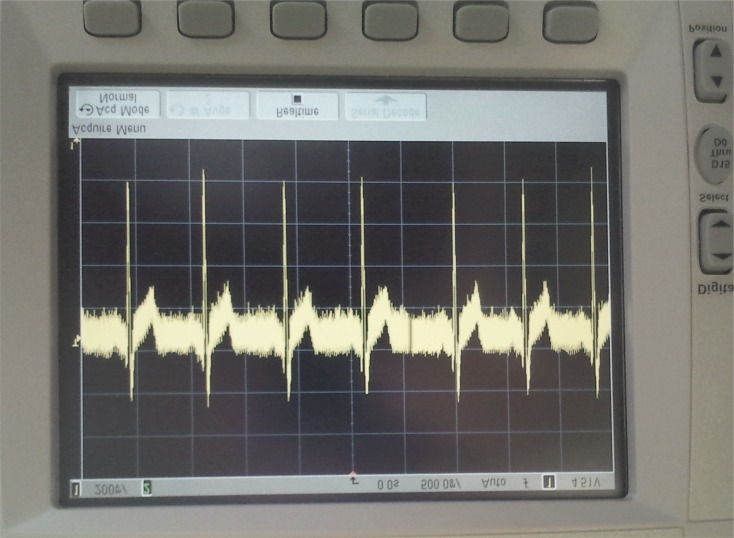
Results for the ECG signal: ECG signal in the electronic node previous to the filtering stage (oscilloscope photo).

**Fig 10:**
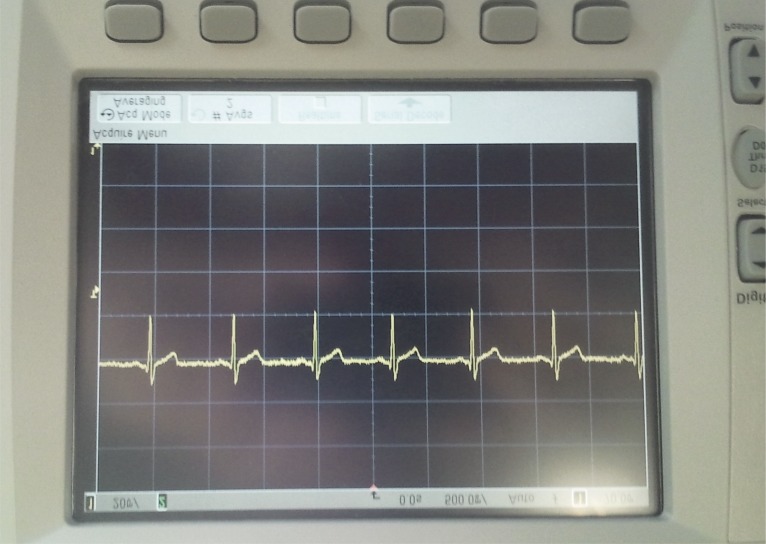
Results for the ECG signal: ECG signal in the electronic node after the filtering stage (oscilloscope photo).

**Fig 11:**
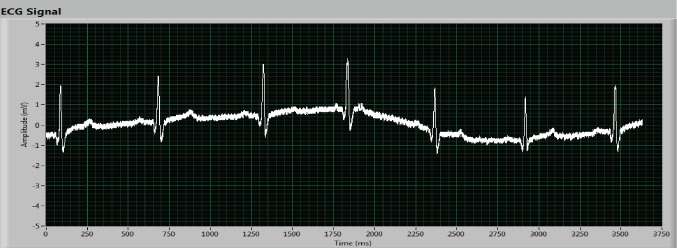
Results for the ECG signal: ECG signal after the wireless transmission (screenshot of the UI designed with LabVIEW).

The device located outside the HC acts as network root and receives the information from the different nodes inside the chamber. The received data is showed on a LabVIEW computer application with a complete User Interface (UI), see Figs 9–11 where real experiments can be seen. This UI has a large number of processing and representation options (Fig 11 partly shows the UI), which allows a detailed analysis of the data collected during the sessions. Each patient has a node to transmit their vital signs and the UI captures and processes the signal data from all nodes. The UI configures the reception port, shows both the raw data and the graphic representation and stores the received data in a plain text file for post-analysis purposes. The developed UI graphically shows and records the vital signs data individually for each patient. However, it could be developed an improved UI where, for instance, a specific vital sign (e.g., the ECG) might be shown and recorded for all the patients inside the HC. Figs 9–11 shows the ECG signal of a patient at different stages: before filtering (Fig 9); after filtering (Fig 10); and the graphical representation shown by the UI after the wireless data transmission (Fig 11).

**Fig 12:**
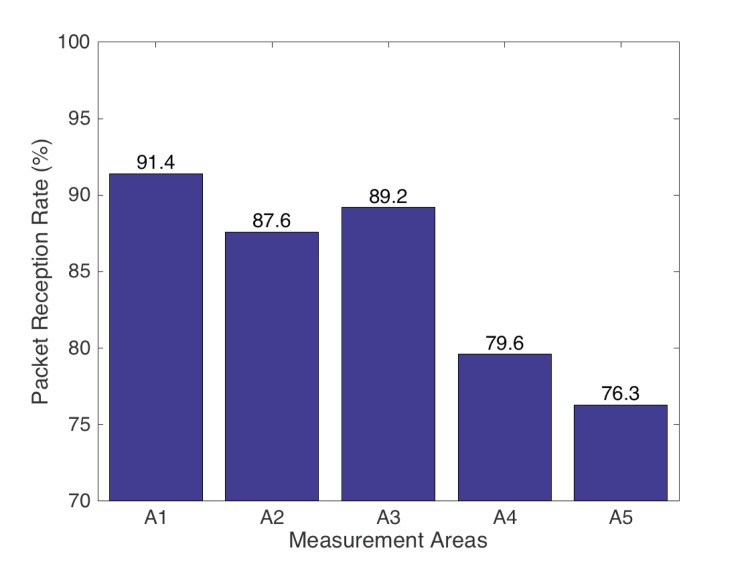
Average reception rate at different locations in the Hospital facilities (values as a percentage of the ZigBee maximum transmission rate of 250 Kbs).

**Fig 13:**
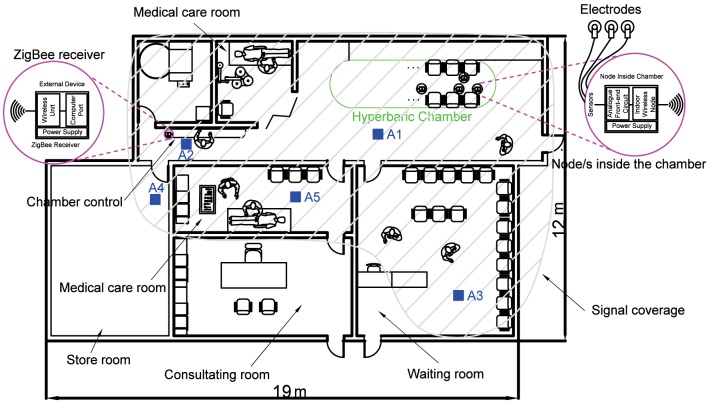
Coverage area for the node inside the hyperbaric chamber.

The coverage area of the proposed wireless system has been experimentally evaluated in the HC environment of the Perpetuo Socorro Hospital in Alicante (Spain). Fig 12 shows the average value of the coverage with the prototype node located inside the HC and the external device located in five different areas, namely {A1, A2, A3, A4, A5}, see Fig 13. The coverage area allows receiving the information in multiple places (shaded area of Fig 13): from the Chamber control to the observation cabin. The results in Fig 12 show that the maximum Packed Reception Rate (PRR) is obtained close to the hyperbaric chamber and near the methacrylate windows, i.e., in area A1. Note that, the weaker the received signal is, the lower the PRR gets.

**Fig 14:**
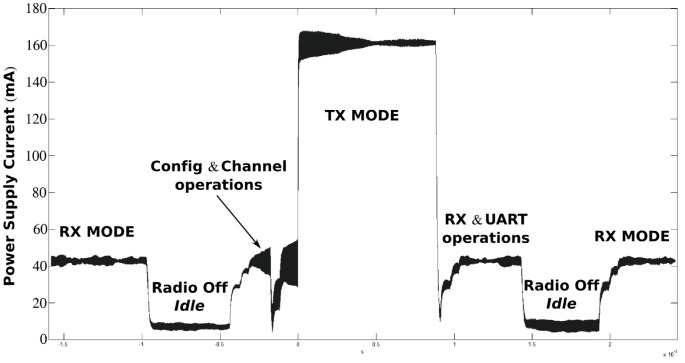
Current supplied to the XBee module.

The power supply of each node consists on a battery block, which must meet the system power requirements and autonomy for the application. To fulfill these requirements, the voltage levels and the maximum currents must be taken into account. On the one hand, the system requires three different voltage values: the maximum positive voltage value (+6V) powers the analogue front-end circuit; the minimum positive voltage value (3,3V) powers the microcontroller and transmitter circuits; and the negative voltage (-6V) is required for the inverter amplifier, to provide a negative output by means of a positive input. On the other hand, the maximum current consumption measured for the nodes is around 160mA. Since the HBO2 treatment duration is estimated around 60–90 minutes, the power supply source for each node has been designed with four AA batteries with a nominal voltage of 1.5V and a capacity of 700mAh. The capacity of the batteries was tested connecting a SHUNT resistor of 4.9 ohms, recording the time until the output voltage falls below the cut-off voltage of the system, which is estimated in 3.8V. In particular, the performed tests showed that the battery supply has an average autonomy of about 6 hours with the conditions mentioned above. Fig 14 shows the current consumption of the XBee module for each stage: transmission mode consumes around 160mA, while reception mode consumes around 45mA.

## Conclusions

This work has presented a ZigBee-based network to obtain a wireless system for monitoring patient's electrical biosignals in hyperbaric chamber applications. The system has been designed with the following objectives:

- Low power consumption in order to reduce the size of the power supply to get a wearable device.
- Low cost due to the price of components used in the implementation.
- Real-time monitoring in order for the medical stuff to observe the electrical biosignals instantly.
- Pressure resistance in order for the devices to support up to 6 atmospheres.

The developed device has been experimentally tested and analyzed in the facilities of the Perpetuo Socorro Hospital (Alicante, Spain) in order to show the effectiveness and advantages of the proposed approach.
